# Depression and suicidal behavior in LGB and heterosexual populations in Serbia and their differences: Cross-sectional study

**DOI:** 10.1371/journal.pone.0234188

**Published:** 2020-06-08

**Authors:** Janko Janković, Vesna Slijepčević, Vladimir Miletić

**Affiliations:** 1 Institute of Social Medicine, Faculty of Medicine, University of Belgrade, Belgrade, Serbia; 2 Institute of Public Health of Belgrade, Belgrade, Serbia; 3 Association for Mental Health Promotion, Belgrade, Serbia; University of Toronto, CANADA

## Abstract

**Background:**

Sexual identity is a significant risk factor for triggering symptoms of depression, as well as for suicide attempts in lesbian, gay and bisexual (LGB) population compared to the heterosexual population. To the best of our knowledge, data on the mental health of LGB persons in Serbia are lacking, and this is the first study to address this problem. The aim of the study was to examine the association between selected determinants and depression, and selected determinants and suicide attempts in LGB and heterosexual populations in the Republic of Serbia, as well as, their differences.

**Methods:**

We conducted a cross-sectional study in 2015 of 264 heterosexual and LGB respondents using the "snowball sampling" method. We used linear regression analyses to investigate the relationship between socio-demographic variables and different sexual identity categories with PHQ-9 scores. We assessed associations between suicide attempts, and socio-demographic characteristics, sexual identity, depression, and suicidal thoughts using logistic regression.

**Results:**

Depression symptoms were higher in female relative to male participants, in participants who were single, divorced or widowed in comparison to currently married, among people with a middle level of education compared to highly educated, and in respondents identified as bisexual or homosexual in comparison to heterosexual. Homosexual and bisexual participants reported suicidal attempts 27 [Odds Ratio (OR) = 27.31] and six times (OR = 6.40) more often than did heterosexual respondents, respectively. Suicide attempts were less frequently reported by single, divorced or widowed participants in comparison to married (OR = 0.25) and those with middle education (OR = 0.38) compared to highly educated.

**Conclusions:**

The present study showed that LGB persons in Serbia have significantly more symptoms of depression and suicide attempts compared to heterosexuals. Public health interventions should focus on the early detection of depression and on overcoming prejudicial and discriminatory attitudes. Also, intervention should emphasize that homosexuality and bisexuality are normal, equal and morally acceptable expressions of human sexuality.

## Introduction

According to the WHO, one in four people will develop a mental health problem in their lifetime, whereas over 300 million people are currently suffering from depression [1]. It is predicted that by 2030 depression will be the most common cause of morbidity in the world, which indicates an ever-growing need for a more systematic approach to mental health improvement and protection [2]. According to a Serbian National Health Survey from 2013, 4.1% of the adult population showed significant symptoms of depression [3]. Depression is the main cause of close to 800.000 suicides annually, making it a serious public health issue worldwide [4]. Suicide is the second leading cause of death among youth and the highest suicide rates are found in vulnerable groups exposed to discrimination [4].

Results from multiple studies indicated that the lesbian, gay, and bisexual (LGB) population is more prone to developing mental health problems, compared to the heterosexual population, because of chronic exposure to damaging effects of homophobia, heterosexism, prejudices, stigma and discriminatory attitudes held by the dominant heterosexual population [5–8]. They also face a higher prevalence of mood and anxiety disorders, higher risk for poor mental health, and higher rates of suicide attempts [5]. 80% of sexual minorities have experienced some kind of harassment in their lifetime, as well as stressful social interactions, which consequently leads to a spectrum of mental health issues [9].

Studies showed that depression is influenced by numerous socio-demographic determinants and is more common in females, young people, those not married or living with a partner, as well as among people with lower educational attainment [10–12]. Conversely, suicide rates are higher in males [12]. The strongest predictor of suicide is a previous suicide attempt [4], whereas the most significant risk factor is an unrecognized or untreated mental health issue [13].

Sexual identity is a significant risk factor for triggering symptoms of depression [9,14], as well as for suicide attempts [15] in LGB compared to the heterosexual population. Studies have found a higher prevalence of suicide attempts among LGB persons, although evidence is not conclusive [7,16]. Young homosexual and bisexual persons have a two times higher risk of suicide attempts than their heterosexual peers [17]. In Serbia, LGB relationships are not legally regulated. Significant physical and verbal violence, as well as, high rates of homophobia are present in public discourse [6]. These factors consequently make LGB relationships and persons nearly invisible.

The non-governmental human rights organization Amnesty International, identified Serbia as a country where national legislation often fails to meet international standards. Further, when legislation does meet international standards, it is often poorly implemented. Government institutions demonstrate a marked lack of will to tackle homophobia and transphobia to protect LGB individuals and organizations from discrimination. Moreover, LGB persons in Serbia face legal challenges not experienced by non-LGB residents. Both male and female same-sex sexual activities are legal, and discrimination on the basis of sexual orientation in areas such as employment, education, media, etc. is banned. Nevertheless, households headed by same-sex couples are not eligible for the same legal protections available to opposite-sex couples [18]. The European Region of the International Lesbian, Gay, Bisexual, Trans and Intersex Association (ILGA-Europe) ranked Serbia 30th in terms of LGBT population rights out of 49 observed European countries [19].

To our knowledge, data are lacking on the mental health of LGB persons in Serbia, and this is the first study to address this problem. Therefore, our aim was to examine the association between different determinants and depression and suicide attempts in LGB and heterosexual populations, as well as, their differences.

## Method

### Study design and sample

This cross-sectional study was conducted in Serbia in 2015 (from June 1 to December 31) and comprised 264 respondents with heterosexual and LGB orientation. The Association for Mental Health Promotion from Belgrade conceived and realized the study using the "snowball sampling" method which is commonly used to access hidden or unreachable populations. This method was chosen because the LGB population is nearly invisible in public discourse and the lack of health centers and organizations devoted to LGB persons means it impossible to conduct the study otherwise. The initial sample was formed by choosing five LGB persons (one man and two women identified as homosexuals and one man and one woman identified as bisexuals) as well as five heterosexual people, who participated in any of the previous projects organized by the Association for Mental Health Promotion and were sensitized to the topic of sexual identities. Initial participants were given online questionnaires to fill out and tasked with recruiting further participants of the same gender and sexual orientation.

The Ethics Committee of the Association for Mental Health Promotion approved the study (decision No. 6/2015, dated May 5, 2015). Prior to filling out the form, all participants provided informed consent to anonymously participate in the study and gave permission to researchers to use the data.

### Instruments

Study participants filled out the following questionnaires:

1. The questionnaire designed specifically for this study which contained information on socio-demographic characteristics (gender, age, marital status, education, employment, monthly income, place of residence), sexual identity, important life events, information about “coming out” to family and friends, questions pertaining to suicidal ideas, and lifetime suicide attempts.
2. The Patient Health Questionnaire–PHQ-9 [20] is a self-assessment questionnaire developed by Pfizer in order to measure symptoms of depression based on DSM-IV criteria (Diagnostic and Statistical Manual of Mental Disorders, 4^th^ edition). It contains nine questions referring to diagnostic criteria rated on the Likert-type scale from 0 (not present at all) to 3 (almost every day).

### Study variables

Based on a literature review [10–12, 21,22], the following independent variables were selected from the database in order to examine their impact on depression symptoms and suicide attempts: demographic variables, including age, gender (male/female), and marital status (categorized as married or living with a partner and single, divorced, widowed); education defined according to the International Standard Classification of Education as high education (college and university degree), middle (three or four years of secondary school), low (no education, incomplete primary school, and primary school); "coming out" to family and friends; variable pertaining to sexual identity categorized as heterosexual, homosexual and bisexual; as well as a variable related to the presence/absence of suicidal thoughts. Monthly income and important life events were not analyzed due to a large number of missing values. Dependent variables were PHQ-9 score to assess the severity of depressive symptoms and past lifetime suicide attempts as a dichotomous variable (yes/no). The question regarding suicide attempts used in this research was: "Have you ever attempted suicide in your lifetime?" The time frame encompassed the entire lifetime of the participants.

### Statistical analysis

Data were analyzed by methods of descriptive statistics and univariate and multivariate linear and logistic regression. To test statistical significance between independent variables and different sexual identities, Pearson’s chi-square test and ANOVA were used. Univariate and multivariate linear regression models were used to investigate the association between socio-demographic variables and different sexual identity categories with PHQ-9 score as the dependent variable. Univariate and multivariate logistic regression was used to assess the association between socio-demographic characteristics, sexual identity, depression and suicidal thoughts with the dependent variable suicide attempts. In analyses with suicidal attempts as the dependent variable, for "suicidal thought" we used the item that consisted of the following questions: "Did you consider suicide in the past six months? Did you have black thoughts? Have you considered making your life meaningless?" The reason to use this item instead of the item #9 of the PHQ-9 ("Thoughts that you would be better off dead, or of hurting yourself in the previous two weeks") was the longer period it refers to (six months vs. two weeks for the item #9 of the PHQ-9 questionnaire). These two items correlated significantly (r = 0.629; p = 0.000), and therefore we entered only one item in the logistic regression model. The threshold for retaining variables in the final multivariate models was p<0.05 in the univariate models. Unstandardized regression coefficients and p values were reported for linear models, and odds ratios (ORs) and 95% confidence intervals (CIs) for logistic models. p<0.05 probability value was taken as a nominal level of statistical significance. All statistical analyses were performed with the IBM SPSS V.20.0 (SPSS Inc, Chicago, Illinois, USA).

## Results

The distribution of sexual identity categories and their differences according to respondent characteristics are shown in Table 1.

**Table 1:** Distribution of sexual identity categories and their differences according to respondent characteristics.

Variables	Heterosexual	Homosexual	Bisexual	*P*-value*
Total n (%)	124 (47.0%)	94 (35.6%)	46 (17.4%)	
Gender, n (%)				
Male	44 (35.5)	59 (62.8)	19 (41.3)	<0.001
Female	80 (64.5)	35 (37.2)	27 (58.7)	
Age, mean (SD)	33.0 (9.6)	32.1 (6.8)	32.5 (6.4)	0.711
Age groups, n (%)				
<20	1 (0.8)	4 (4.3)	0 (0)	0.111
21–29	48 (38.7)	29 (30.9)	17 (37.0)	
30–39	48 (38.7)	47 (50.0)	22 (47.8)	
40–49	20 (16.1)	14 (14.9)	6 (13.0)	
>50	7 (5.6)	0 (0)	1 (2.2)	
Education, n (%)				
Middle education	26 (21.0)	39 (41.5)	29 (63.0)	<0.001
High education	98 (79.0)	55 (58.5)	17 (37.0)	
Marital status, n (%)				
Single, divorced, widowed	59 (47.6)	64 (68.1)	27 (58.7)	0.001
Married/living with a partner	65 (52.4)	30 (31.9)	19 (41.3)	
Employment, n (%)				
Unemployed	38 (30.6)	59 (62.8)	25 (54.3)	<0.001
Employed	86 (69.4)	35 (37.2)	21 (45.7)	
Residence, n (%)				
Belgrade	76 (61.3)	51 (54.3)	16 (34.8)	0.001
Vojvodina	31 (25.0)	34 (36.2)	15 (32.6)	
Inner Serbia	17 (13.7)	9 (9.6)	15 (32.6)	
Coming out to family, n (%)				
To everyone		29 (30.9)	11 (23.9)	<0.001
To some family members		25 (26.6)	2 (4.3)	
To no one		40 (42.6)	33 (71.7)	
Coming out to friends				
To everyone		32 (34.0)	5 (10.9)	<0.001
To some		56 (59.6)	16 (34.8)	
To no one		6 (6.4)	25 (54.3)	
Satisfaction with coming out to family		.		
Satisfied		23 (42.4)	10 (75.6)	<0.001
Unsatisfied		31 (57.6)	3 (24.4)	
Satisfaction with coming out to friends				
Satisfied		73 (82.6)	12 (57.8)	0.001
Unsatisfied		15 (17.4)	9 (42.2)	
PHQ-9, average score (SD)	7.7 (5.6)	8.8 (6.3)	13.0 (5.8)	<0.001
Suicidal thoughts^†^				
Yes	19 (15.3)	10 (10.6)	4 (8.7)	0.405
No	105 (84.7)	84 (89.4)	42 (91.3)	
Suicidal attempts				
Yes	6 (4.8)	31 (33.0)	7 (15.2)	<0.001
No	118 (95.2)	63 (67.0)	39 (84.8)	

*According to Chi-square test and ANOVA where appropriate.

^†^In the previous six months period.

SD: Standard deviation; PHQ-9: Patient Health Questionnaire.

In total 264 participants completed the questionnaire, out of which 47% were heterosexual, 35.6% homosexual, and 17.4% identified as bisexual. Of the total number of participants, 46.2% were men, and 53.8% women. Participants' age ranged from 17 to 77 (mean age 32.62; SD = 8.15). The highest percentage of participants with high education (79%) and those who are married or living with a partner (52.4%) were found among heterosexual participants. The average score of depression symptoms was highest in bisexual participants (13.0±5.8) compared to homosexual (8.8±6.3) and heterosexual participants (7.7±5.6), while those identified as homosexual most frequently reported suicide attempts (33%). The majority of homosexual respondents said they came out to family (57.5%) or friends (93.6%). Of those, 42.4% and 82.6% were satisfied with how it went when they came out to family and friends, respectively. Only 28.2% of bisexual respondents came out to family, and 45.7% came out to friends. Two thirds (75.6%) and more than a half (57.8%) of bisexuals were satisfied with how it went when they came out to family and friends, respectively. Except for age and suicidal thoughts, statistically significant differences were found between all variables and categories of sexual identity (Table 1).

Table 2 shows the distribution of PHQ-9 scores according to sexual identity categories.

**Table 2:** Distribution of PHQ-9 scores according to sexual identity.

Symptom severity	PHQ-9 score	Total	Heterosexual	Homosexual	Bisexual
		N (%)	N (%)	N (%)	N (%)
No symptoms	0	5 (1.9)	4 (3.2)	1 (1.1)	0 (0.0)
Minimal	1–4	82 (31.1)	39 (31.5)	36 (38.3)	7 (15.2)
Mild	5–9	76 (28.8)	41 (33.1)	26 (27.7)	9 (19.6)
Moderate	10–14	36 (13.6)	23 (18.5)	6 (6.4)	7 (15.2)
Moderately severe	15–19	45 (17.0)	14 (11.3)	17 (18.1)	14 (30.4)
Severe	20–25	20 (7.6)	3 (2.4)	8 (8.5)	9 (19.6)

PHQ-9: Patient Health Questionnaire.

PHQ-9 score ranged from 0 to 25, with a mean of 9.02 (SD = 6.17). The highest number of participants reported minimal (31.1%) or mild level of depressive symptoms (28.8%), whereas bisexual participants most frequently showed symptoms of moderately severe or severe depression (Table 2).

Results of univariate and multivariate linear regression analyses of PHQ-9 scores are shown in Table 3.

**Table 3:** Associations of PHQ-9 scores with socio-demographic characteristics and sexual identity.

Variables	Univariate linear regression		Multivariate linear regression	
	B	*P*	B	*P*
Gender	1.986	0.009	3.291	<0.001
Age	0.098	0.035	0.076	0.075
Marital status	2.781	<0.001	2.995	<0.001
Education	3.126	<0.001	1.693	0.029
Sexual identity	2.351	<0.001	2.023	<0.001

B, unstandardized regression coefficient.

According to the multivariate linear regression analysis, PHQ-9 scores are higher in women (p<0.001), in respondents who are single, divorced or widowed in comparison with currently married (p<0.001), in participants with middle education compared to highly educated (p = 0.029), as well as among bisexual and homosexual participants in comparison to heterosexual ones (p<0.001).

Associations of suicide attempts with socio-demographic determinants, sexual identity, depression, and suicidal thoughts are shown in Table 4.

**Table 4:** Associations of lifetime suicide attempts with socio-demographic determinants, sexual identity, depression and suicidal thoughts.

Variable	Univariate logistic regression	Multivariate logistic regression
	OR (CI)	OR (CI)
Gender		
Male	1	1
Female	2.07 (1.04–4.11)*	3.14 (1.25–7.88)*
Age	1.01 (0.97–1.05)	
Marital status		
Married/living with a partner	1	1
Single, divorced, widowed	0.33 (0.17–0.64)^†^	0.25 (0.10–0.64)*
Education		
High education	1	1
Middle education	0.41 (0.19–0.89)*	0.38 (0.15–0.97)*
Sexual identity		
Heterosexual	1	1
Homosexual	9.68 (3.83–24.43)^†^	27.31 (9.31–80.16)^†^
Bisexual	3.53 (1.12–11.14)*	6.40 (1.88–22.92)*
Depression (PHQ-9 score)	0.98 (0.93–1.04)	
Suicidal thought^‡^		
No	1	1
Yes	1.73 (0.73–4.15) *	4.30 (1.46–12.66)*

*p<0.05

^†^p<0.001

^‡^In the previous six months period.

OR, Odds Ratio; CI, Confidence Interval.

Suicide attempts are three and four times more likely reported by female participants (OR = 3.14) and persons having suicidal thoughts (OR = 4.30), respectively, whereas homosexual and bisexual participants compared to heterosexual participants attempted suicide 27 (OR = 27.31) and six times more often (OR = 6.40), respectively. On the other hand, suicide attempts are less likely reported by participants who are single, divorced or widowed (OR = 0.25) in comparison to currently married/living with a partner, or those with middle education (OR = 0.38) compared to highly educated. Because we expected that depression might be a possible predictive factor for suicide attempts, we added depression in the multivariate logistic regression model and coefficients did not differ substantially (data not shown).

## Discussion

Our study found that LGB persons reported significantly more symptoms of depression than their heterosexual peers. A US study by Koch and Ross [23] found that 56.7% of lesbians and 53.2% of bisexual women had depression as a diagnosis compared to only 42.1% of heterosexual women. A large Australian study [24] conducted in 2006 among 5476 LGBT persons reported that prevalence of symptoms of depression was very high, that is, 70% of male and 80% of female participants reported the presence of depressive symptoms during a lifetime. King et al. [25] demonstrated that depression rates in the LGB population are 1.5 higher than among heterosexuals. Higher levels of symptoms of depression among LGB persons could be explained by feeling that they present a burden in other people's lives [26]. An American study [27] showed that bisexuals have significantly higher levels of depression compared to both homosexual and heterosexual persons, probably due to feeling of low community affiliation. Shilo and Savaya [28] noted higher levels of psychological distress in bisexuals and explained it not only by lack of belonging to a community, but also by lack of social and family support and higher degrees of internalized homophobia.

Further, our results indicated that non-heterosexual identity coupled with suicidal thoughts present significant predictors of suicide attempts. Suicide attempts were six times more common in bisexual and 27 times in homosexual respondents compared to heterosexuals. Numerous studies showed that suicide attempts in the LGB population tend to be two to 17 times more frequent than in heterosexuals [25,29,30]. One Switzerland study [31] pointed out three main reasons to explain the disproportionately high suicide attempt rates among gay men: problems in intimate relationships, difficulties accepting their homosexuality, and family rejection. According to Kerr [26], the key mechanism that explains the frequency of suicidal ideation among young LGB persons contains elements such as coming out in public coupled with a feeling of being a burden to others due to their sexuality. Marshal et al. [32] found that the presence of negative discriminatory experiences generates significant differences in suicide rates and depression between sexual minorities and heterosexuals. Lack of support, family rejection as a result of coming out in conservative environments, as well as violence are key factors in many studies that influence suicide attempts among young LGB persons [33–36]. According to a study by Hill and Pettit [34] social isolation and lack of social support are contributing factors to suicidal ideation, increasing the chance of suicide attempts by three times.

Many prospective studies have documented the relationship between depression and subsequent suicide attempts [37–39]. Analyses of the data from the National Longitudinal Study of Adolescent Health in the USA, revealed that the association between depression and suicide attempts was stronger among non-LGB respondents than among LGB respondents [40]. However, we did not find any association between depression and suicidal attempts, which is in accordance with several other studies [41–43]. These inconsistencies raise questions about whether and to what degree depression confers risk for suicidal behaviors [22]. One of the explanations why depression was not a significant predictive factor for suicide attempts in our sample could be that self-reported measures of depression, like PHQ-9 used in our and other epidemiological studies, are screening questions which serve only as a proxy of clinical depression, and may result in measurement error. The gold standard for a research diagnosis of depression should be, for example, the Structural Clinical Interview, a time-consuming and expensive to implement. In spite of relatively similar depression scores in heterosexuals and homosexuals in our study, suicide attempts were much higher (27 times) among homosexuals. This could be explained by a number of factors to which homosexuals are exposed: problems in intimate relationships, family rejection, social isolation, lack of social support, victimization, workplace discrimination, and marginalization such as bullying, harassment, and physical violence [31,32].

Our results showed that symptoms of depression were significantly more frequent in females than males, which is in accordance with many other studies [3,12,27,44] supporting the notion that the female gender is a risk factor for depression. Higher levels of depression in women are explained by synergistic effects of psychological, cultural and social factors. Psychological factors can include specific attachment patterns, personal constructions of gender roles, biological differences, and power dynamics of gender differences on a macro level. Equally important are social and cultural contexts which tend to place women in a less favorable position, as well as the fact that women’s social roles are constructed in such a way that they perform far more social duties [45]. From a biological standpoint, the cyclical nature of hormonal changes makes women prone to an augmented stress response that can eventually lead to depression [46]. A study by Baams et al. [27] indicated that levels of depression among LGB youth differed across gender and sexual identities. Bisexual girls reported higher levels of depression and perceived burdensomeness than lesbian girls and gay boys. These results suggest that the feeling of being a burden to others is a critical mechanism in explaining higher levels of depression and suicidal ideation among LGB youth.

Our finding that there is a threefold increase in suicide attempt risk for female participants compared to males was also documented in the American [47] and the Caribbean study [12]. One Ethiopian study found 1.63 increased odds of suicidal behavior (OR = 1.63; 95% CI: 1.45–4.76) in females, compared to males [48] while in a Chinese study [49] the risk of suicidality in women was more than twice that in men (OR = 2.62; 95% CI: 1.45–4.76). Globally, rates of suicidal thoughts and suicide attempts are higher among women, while men have high rates of suicide [12,50]. This may be explained by differences in gender roles and may be consistent with a more complex and culturally specific understanding of masculinity, power, and powerlessness [50]. In countries like China, for example, suicide rates of young women are higher compared to men, because the act of suicide is considered an act of powerlessness and weakness, which implies cultural diversity in gender patterns and interpretation of suicidal behavior [50]. Differences between men and women are frequently explained by the choice of suicide method; men tend to choose deadlier methods, and suicide attempts are more aggressive with a pronounced desire to die [47].

Regarding marital status, our findings are consistent with the results of many other studies [12,47], where people who reported being single, divorced or widowed had a greater probability of developing depression, but a lower risk of suicide attempts than currently married [10,47]. This could be explained by the protective effects of community in terms of psychological support, sharing costs of life, a healthier lifestyle and consequently lower level of stress. Persons reported being single or experiencing a high level of stress related to divorce or death of their partner, had a greater probability of developing depression [10].

In our study, participants with middle education showed a higher risk of depression, but a lower risk of suicide attempts than those with high education. This finding is in contrast with the studies supporting the protective effect of high education or cohabitation on depression and suicide attempts [10,47]. One reason may be our sampling method and the way in which participants recruited other similar participants. A Caribbean systematic review study by Brown et al. [12] which examined an association between socio-demographic determinants with depression and suicidal behavior showed that persons with lower educational attainment have greater chances of developing depression, compared to those with higher education. Lower educational levels are related to the feeling of social inadequacy that may lead to the development of depression [47]. Persons with lower education can feel helpless and socially trapped which, according to some psychological theories, contributes to the development of psychiatric disorders [51].

This study showed that participants were more likely to turn to friends than family and were satisfied with coming out to friends. Most studies of friendship have assessed its effects on mental and physical health outcomes [52,53], but not on suicidal behavior. Marver et al. [21] explored the effect of friendship on suicide attempt risk during one-year follow-up of 132 adults presenting with a major depressive episode and concluded that impaired friendship predicted a greater risk of suicide attempt. The explanation is that friendship has a potentially bidirectional relationship with depression, and that its effect on suicidal behavior occurs through its relationship with depression [21].

The mental health of LGB persons in Serbia is still uncharted territory and to the best of our knowledge, this is the first study to document predictors of depression and suicide attempts in such population. Also, several drawbacks should be briefly stressed. The snowball sampling method is a limitation of our study in a way that we are not sure if the results can be generalized to the entire population. For example, this study did not include those LGB persons with low education, since people that participated in our study move in similar social strata. Also, the PHQ-9 is a questionnaire that measures symptoms of depression and does not diagnose the disorder, which requires a more complex psychiatric check-up and proper psychiatric history. All questions were self-reported and may be subject to recall bias. Nevertheless, this study is a good basis for further research, using more detailed questionnaires related to suicidal behaviors and psychiatric history. Given the worrying levels of prejudice, stigma, discrimination, and violence against the LGB population in Serbia, the most challenging issue facing future research is how to reach this population. As explained above, snowball sampling, which is a method of choice for hidden populations, such as LGB, has some limitations. On the other hand, probability sampling, although it allows generalizations, has limited use in LGB research in Serbia because of the relatively small size of the hard-to-find population, the lack of research funding, and the sensitivity of questions related to sexual behavior and gender expression. We think that a time-locating sampling would be the best method for future research on the Serbian LGB population. The principle is to reach individuals in places and at times where and when they gather. For example, men who have sex with men meet in gay venues at certain times of the day. This sampling approach uses multiple stages of data collection to increase the likelihood of developing a representative sample of the target study population.

The main results of our study suggest that there is a strong relationship between sexual identity and suicidal thoughts and depression symptoms and lifetime suicide attempts in LGB and heterosexual populations. Considering higher depression scores and higher numbers of suicide attempts in LGB persons, it is important to work on overcoming prejudices and discriminatory attitudes towards them in both public and private spheres and to support a gay-affirmative approach that allows approaching the person in a supportive and understanding way. Mental health professionals and society at large have to work more in order to accept variations of human sexuality as equal and morally acceptable. Specifically designed public health interventions for LGB people are warranted not only because they are at higher risk for developing depression, but also because of the overall negative social climate. Public health activities should be directed on early detection of depression in LGB people, and towards changing prejudicial and discriminatory attitudes in public discourse.
